# The Distribution of High-Risk Human Papillomaviruses Is Different in Young and Old Patients with Cervical Cancer

**DOI:** 10.1371/journal.pone.0109406

**Published:** 2014-10-08

**Authors:** Mariano Guardado-Estrada, Eligia Juárez-Torres, Edgar Román-Bassaure, Ingrid Medina-Martinez, Ana Alfaro, Rosa Elba Benuto, Michael Dean, Nicolás Villegas-Sepulveda, Jaime Berumen

**Affiliations:** 1 Unidad de Medicina Genómica, Facultad de Medicina, Universidad Nacional Autónoma de México/Hospital General de México, México, D.F. México; 2 Servicio de Oncología, Hospital General de México. México, D.F. México; 3 Academia Mexicana de Dermatología, México, D.F. México; 4 Laboratory of Experimental Immunology, National Cancer Institute, Frederick, Maryland, United States of America; 5 Departamento de Biomedicina Molecular, Centro de Investigación y Estudios Avanzados del Instituto Politécnico Nacional. México, D.F. México; 6 Departamento de Medicina Experimental, Facultad de Medicina, Universidad Nacional Autónoma de México. México, D.F. México; Penn State University School of Medicine, United States of America

## Abstract

Despite numerous human papillomavirus (HPV) frequency studies in women with cervical cancer (CC), little is known of HPV frequency trends according to patient age. In this work, we compare the mean age and frequency distribution by age of CC patients positive for different HPVs. This study included 462 CC patients. HPVs were detected by PCR and typed using DNA sequencing. A total of 456 patients (98.7%) were positive for HPV: 418 (90.5%) had single and 38 (8.2%) had double HPV infections. HPV16 (46.5%), HPV18 (10.4%), HPV45 (6.7%), and HPV31 (4.1%) were the most frequent viral types in single-infected patients. The mean ages of single-infected patients with HPV16 (49.2±13.3), HPV18 (47.9±12.2), HPV45 (47.9±11.7), or HPV39 (42.6±8.9) were significantly lower than the mean ages of patients singly (53.9±12.7; *p*<0.001, t-test) or doubly (55.4±12.7; *p*<0.05, t-test) infected with the remaining HPVs. Three different trends were identified: one for HPV16, another for HPVs18/45/39, and a third for the rest of HPVs. The frequency trend of HPV16 shows two peaks. The first (63.2%) was found in the youngest women (≤35 years), followed by a decreasing trend until the age of 55–60 years (31.1%). The second peak arose at 61–65 years (52.5%), followed by a decreasing trend. The trend for HPVs18/45/39 declined from the youngest (19.3%) to the oldest (>70 years; 12.8%) women. In contrast, the trend for the remaining HPVs increased from the youngest (15.8%) to the oldest (46.2%) women. Unlike other life-style factors, low-risk sexual behavior was associated with late onset of CC independent of low-oncogenic HPV types (p<0.05, Wald chi-square statistic). The data indicate that most CCs in young women depend on the presence of high-oncogenic HPVs. In contrast, almost half of CCs in older patients had low-oncogenic HPVs, suggesting they could depend on the presence of other factors.

## Introduction

Cervical cancer (CC) is the third most common cancer in women worldwide, affecting 500,000 individuals each year, and it is the leading cause of cancer death among women in developing countries [1]. The human papilloma virus (HPV) is the main causal factor for the development of CC, and HPV is found in nearly 100% of these tumors [2], [3]. HPV16 is the viral type detected most frequently worldwide; it is found in approximately 50% of CC cases, followed by HPV18, HPV45, and HPV31 [3]. HPV prevalence varies in healthy women according to age. In most studies, the highest peak is seen in younger women (<25 years old), then a decreasing trend with age is observed. In contrast, the distribution of CC follows a standard curve, with a maximum peak around 50 years old. Therefore, half of CC cases are diagnosed in younger (pre-menopausal) women and half in post-menopausal women [4], [5]. It has been reported that most CC arises 15–20 years after initial HPV infection [6]. According to the frequency distribution of HPVs in healthy women, these latency periods could explain most of the CC in young and middle-aged, but not elderly women [7]. These data suggest that CC carcinogenesis events in elderly patients could be different than in young patients. Although the mean age of CC patients infected with HPV16, 18 or 45 is lower than that of patients infected with other HPVs [8], [9], [10], [11], little is known of the prevalence and HPV trends in different age groups in women with CC. Therefore, it is important to investigate whether the same HPV types are involved in both groups of patients. In this work, we analyzed the distribution of HPV types according to patient age in a sample of 462 women with CC diagnosed in Mexico City.

## Results

### Age Description of CC Patients

**Figure 1:**
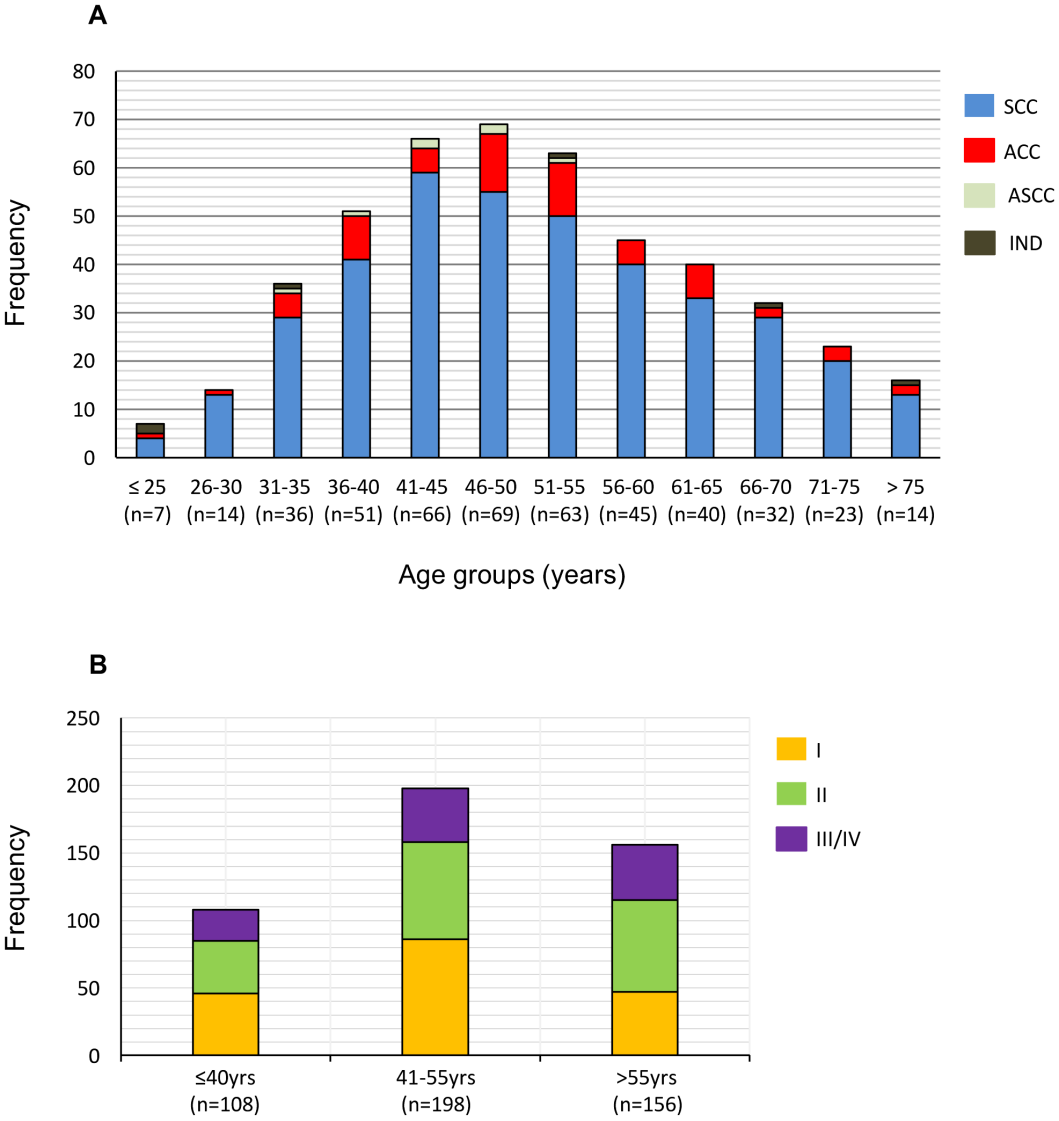
Age distribution of patients with cervical cancer (CC). The age distribution of CC patients divided into 5-year intervals according to tumor histological type (A). The frequency of patients by age, divided into three groups: <41 years, 41–55 years, and>55 years, according to FIGO staging (B). IND, undifferentiated; ASCC, adenosquamous cell carcinoma; ACC, adenocarcinoma; SCC, squamous cell carcinoma.

Age varied widely among CC patients, ranging from 22 to 89 years old (mean  =  50.6±13.0, n = 462). The frequency distribution into 5-year intervals shows one peak at 46–50 years interval. However, the distribution did not follow a normal distribution, but was slightly skewed to the right (Figure 1A). Most CC patients were concentrated in the ranges between 41 and 55 years (n = 198, 42.9%), 108 (23.4%) in the lower ranges (≤40 years), and 156 (33.8%) in the higher ranges (>55 years) (Figure 1A). Although the mean (50.8 vs. 50.6 years) and the median (49 years) age of patients with SCC (n = 386) and ACC (n = 63) were similar, the SCC patients peaked one interval before the ACC patients (41–45 vs. 46–50 years). The skewed distribution of the whole sample is explained by the higher frequency of CC stages II and III/IV in the older than in the younger group (Figure 1B). Interestingly, 30.1% of CC diagnosed in the older patients was FIGO I, indicating that at least this percentage of tumors was initiated in patients older than 55 years.

### Frequency of HPV Types in CC

Of the 462 CC cases analyzed, 456 (98.7%) were positive for at least one HPV, 418 (90.5%) had single, and 38 (8.2%) had double HPV infections. HPV16 was the most frequent viral type in single infections (46.5%), followed in decreasing order by HPV18 (10.4%), HPV45 (6.7%), HPV31 (4.1%), HPV59 (3.2%), HPV58 (2.8%), HPV33 (2.8%), HPV51 (2.6%), HPV56 (1.7%), HPV52 (1.5%), HPV35 (1.1%), HPV39 (1.1%), HPV11 (1.1%), and other HPVs with a frequency lower than 1% (Table 1). There were 11 (2.4%) women with CC that were infected with single low-risk HPVs, including HPV6 (n = 1), HPV11 (n = 5), HPV42 (n = 1), HPV61 (n = 1), and HPV69 (n = 3). As in single infections, HPV16 was the most frequent viral type (4.8%) in double infections, followed by HPV18 (3.0%) (Table 1). As expected, the most common double infection was HPV16 with HPV18 (1.7%). However, three groups of patients with double HPV infections were identified. The first group was infected with any two of HPV16, HPV18, or HPV45; the second group with HPV16, HPV18, or HPV45, plus other HPVs; and the third group with HPVs other than HPV16, HPV18, or HPV45 (Table 2). Although most of the double infections in the third group included at least one high-risk HPV, two patients presented infections with non-high-risk HPVs HPV64/HPV34 and HPV85/HPVCand8. The cumulative frequency of the four vaccine-related HPVs (HPV16, HPV18, HPV31, HPV45), including single and double infections, was 74% (342/462).

**Table 1:** Frequency of single and double HPV infections in cervical cancer patients (n = 462).

HPV type	Frequency of HPV types: n (%)		
	Single infection	Double infection	Total
HPV16	215 (46.5%)	22 (4.8%)	237 (51.3%)
HPV18	48 (10.4%)	14 (3%)	62 (13.4%)
HPV45	31 (6.7%)	2 (0.4%)	33 (7.1%)
HPV31	19 (4.1%)	3 (0.6%)	22 (4.8%)
HPV58	13 (2.8%)	3 (0.6%)	16 (3.5%)
HPV59	15 (3.2%)	1 (0.2%)	16 (3.5%)
HPV33	13 (2.8%)	1 (0.2%)	14 (3%)
HPV51	12 (2.6%)	1 (0.2%)	13 (2.8%)
HPV56	8 (1.7%)	1 (0.2%)	9 (1.9%)
HPV52	7 (1.5%)	1 (0.2%)	8 (1.7%)
HPV6	1 (0.2%)	5 (1.1%)	6 (1.3%)
HPV53	3 (0.6%)	2 (0.4%)	5 (1.1%)
HPV69	3 (0.6%)	2 (0.4%)	5 (1.1%)
HPV35	5 (1.1%)	0 (0%)	5 (1.1%)
HPV39	5 (1.1%)	0 (0%)	5 (1.1%)
HPV11	5 (1.1%)	0 (0%)	5 (1.1%)
Other HPVs^a^	15 (3.2%)	18 (3.9%)	33 (7.1%)
Negative	-	-	6 (1.3%)
All HPV+	418 (90.5%)	38 (8.2%)	456 (98.7%)

a Includes HPV68, HPV51Like, HPV66, HPV26, HPV39Like, HPV42, HPV61, HPV70, HPV73, HPV82, HPV82Like.

**Table 2:** Mean age of cervical cancer patients according to HPV type (n = 462).

HPV type	Mean ± S.D.^a^ (n)
**Single infection**	
HPV16	49.2±13.3 (215)
HPV18	47.9±12.2 (48)
HPV45	47.9±11.7 (31)
HPV31	50.6±10.8 (19)
HPV59	54.3±11.5 (15)
HPV58	55.9±11.7 (13)
HPV51	54.7±12.8 (12)
HPV33	58.2±15.9 (13)
HPV56	58.4±13.9 (8)
HPV52	57.6±10.4 (7)
HPV35	46.4±9.8 (5)
HPV11	47.4±18.1 (5)
HPV39	42.6±8.9 (5)
Other HPVs^b^	53±13.2 (22)
All	50.2±13 (424)
**Double infections**	
Two of any HPV16,18 or 45	47.6±6.7 (9)
Any of HPV16, 18 or 45 plus other HPVs	54.2±13.5 (20)
Others than HPV16,18 or 45	65.8±8.4 (9)
All	55.4±12.7 (38)
HPV negative	50.2±12.6 (6)

a. S.D.  =  standard deviation.

b. Other HPVs included HPV6, HPV26, HPV39Like, HPV42, HPV51Like, HPV53, HPV61, HPV66, HPV68, HPV69, HPV70, HPV73, HPV82, HPV82Like.

The mean±S.D.(n) in the whole sample was 50.6±13.0 (462)

### Analysis of HPV Types by Age

The mean age of the patients with single HPV infections was 50.2±13.0 years; however, a great range of variation was seen among the patients according to viral types: from 42.6 years for HPV39 to 58.4 years for HPV56 (Table 2). Although patients infected with HPV18 (47.9±12.2 years, n = 48) and HPV45 (47.9±11.7 years, n = 31) were slightly younger than those infected with HPV16 (49.2±13.3 years, n = 215) and HPV31 (50.6±10.8 years, n = 19), these differences were not statistically significant (p>0.05, t-test). However, when these groups were compared with patients singly infected with the remaining viral types (54±13.1 years, n = 105), all comparisons but one (HPV31), were statistically significant (p<0.01, t-test). Therefore, HPV31-positive patients behaved more like the patients positive for HPVs other than HPV16, HPV18, or HPV45. Notably, the mean age of patients positive for HPV11, HPV35, and HPV39 was similar to patients positive for HPV16, HPV18, and HPV45, but only the mean of HPV39 patients was significant lower (p<0.05, t-test) than the mean of patients positive for HPVs other than these viruses (Table 2). Regrouping the patients positive for HPV39 with patients positive for HPVs 16, 18, and 45, the mean age of this group was 48.7±12.9 years (n = 299) and the mean age of patients infected with the remaining single viral types was 53.9±12.7 years (n = 119) (p = 1.7×10^−4^, t-test).

Remarkably, the mean age of the patients with double infections was higher than those with single infections (55.4±12.7 years, n = 38; vs. 50.2±13.0 years, n = 418) and this difference was statistically significant (p<0.05, t-test) (Table 2). However, there were differences in the mean age among the three groups of patients with double infections (Table 2).

**Figure 2:**
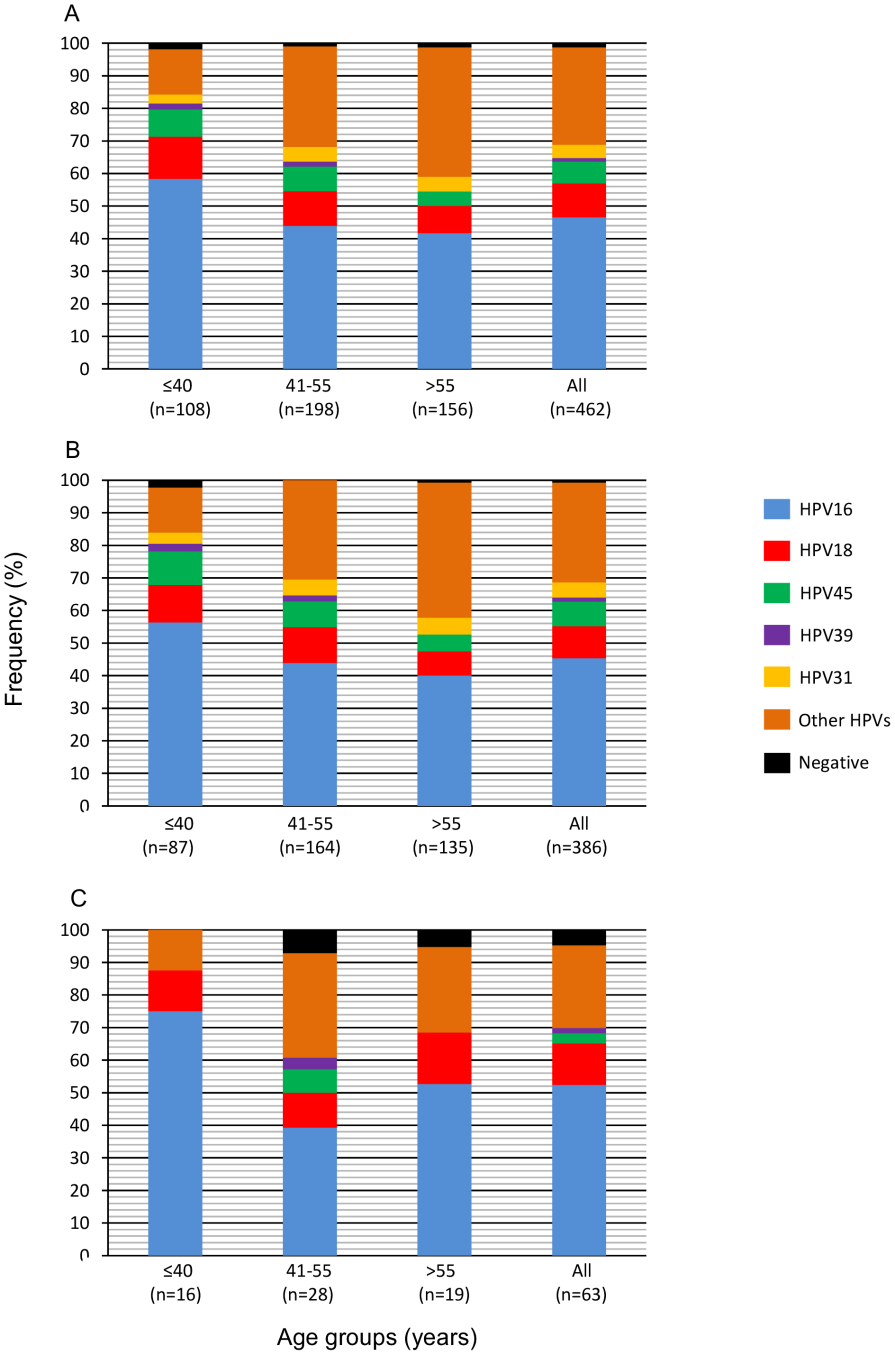
Frequencies of different HPV types in CC patients according to age. The figure shows the relative frequency (%) of HPV types in CC patients grouped by age in ≤40, between 41 and 55 and>55 years old. Panel A included the patients of the whole sample, panel B patients with Squamous Cell Carcinomas, and panel C included patients with Adenocarcinomas. Bars labeled as HPV16, HPV18, HPV45, HPV31 and HPV39 include only single infections. Other HPVs group includes single infection of HPV types 6, 11, 26, 33, 35, 42, 51, 52, 53, 56, 58, 59, 61, 66, 68, 69, 70, 73, 82, 39-like, 51-like, 82-like and all double infections.

The HPV frequencies were studied in patients divided into three age groups: young patients (≤40 years), middle-aged patients (41–55 years), and older patients (>55 years) (Figure 2A). Interestingly, the frequency of HPVs 16, 18, 45, and 39 declines from the younger to the older group, although only the trend for HPV16 was statistically significant (p<0.01, Cochran-Armitage trend test). The HPV16 frequency declines 16.7% from the younger (58.3%) to the older group (41.7%; p = 0.004, chi-square test) (Figure 2A). The pooled frequency of single HPV16, HPV18, HPV45, or HPV39 infections in younger patients was 81.5% (Figure 2A), whereas in the middle-aged and older patients, it decreased to 63.6% and 54.5% (p<0.0001, Cochran-Armitage trend test), respectively. In contrast, the pooled frequency of the other single HPV infections, including HPV31, and all double infections, increased from the younger (16.7%) to the older (44.2%) group (p<0.0001; Cochran-Armitage trend test) (Figure 2A).

**Figure 3:**
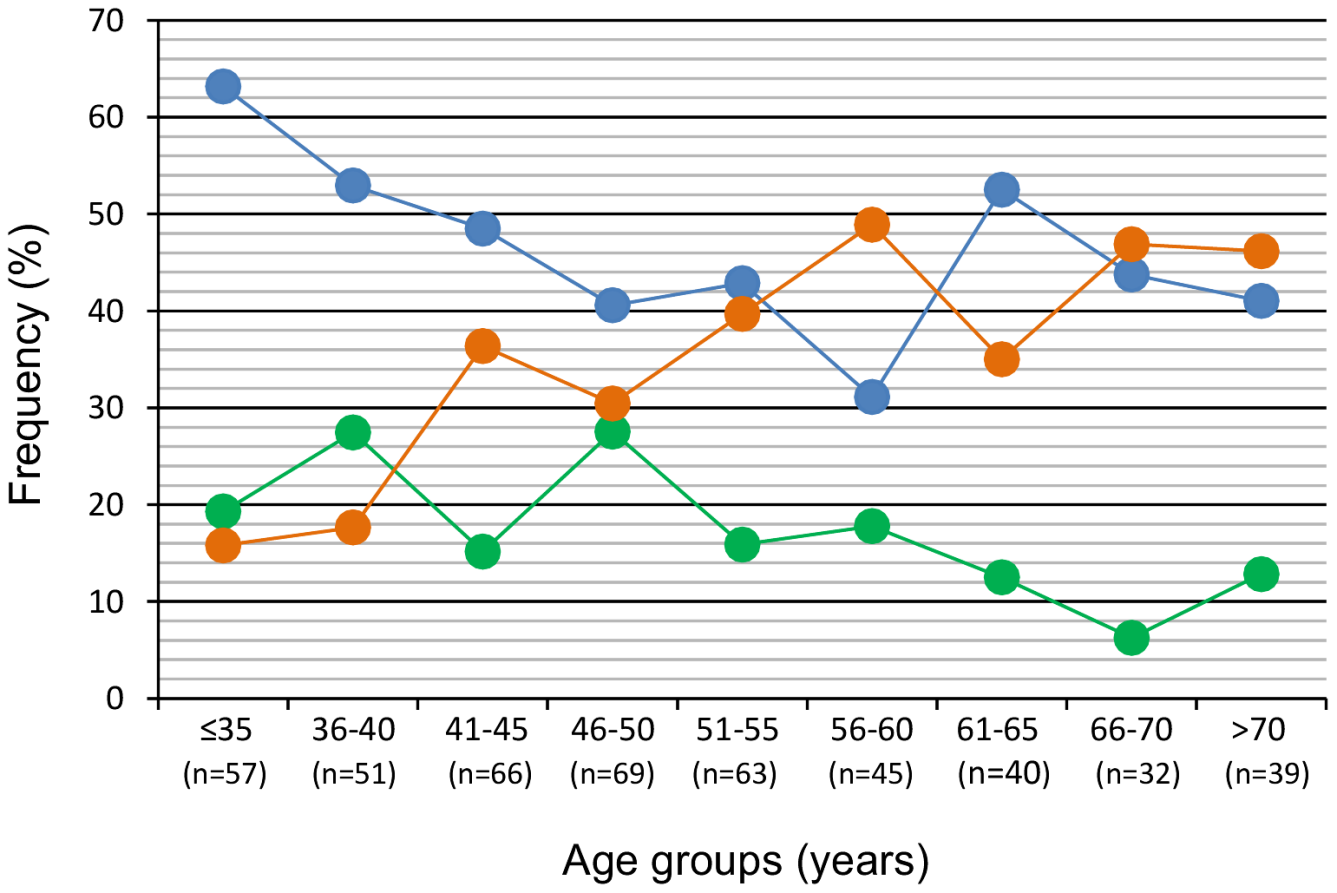
HPV types distribution by 5-years age intervals in the whole sample of CC patients. The figure shows the frequency distribution of HPV16 single infections (blue circles), pooled frequency of HPV18, HPV45, and HPV39 single infections (green circles), and the pooled frequency of other HPV types (see Figure 2) plus HPV31 and all double infections (orange circles) over 5-years age intervals in the whole CC patients (n = 462).

To observe these trends in more detail, the viral type frequency was also investigated in patients grouped by age into 5-year intervals (Figure 3). In the case of HPV16, the frequency had a maximum peak (63.2%) at ≤35 years, and gradually decreased until 56–60 years (31.1%); then a second peak arose (52.5%) at 61–65 years and decreased again afterwards (Figure 3). The first part of the curve had a significant negative correlation (r = −0.899, p = 0.017, Spearman correlation). The pooled frequency of HPV18, HPV45, and HPV39 showed a significant decay from the younger (19.3%) to the older (12.8%) patients (r = −0.68, p = 0.032, Spearman correlation). In contrast, the pooled frequency of single infections with HPVs other than HPVs 16, 18, 45, or 39, plus double infections, showed an increasing trend from younger (15.8%) to older (46.2%) women (r = 0.75, p = 0.01, Spearman correlation).

### HPV Frequency According to Tumor Histology and FIGO Stage

The HPV type frequencies were very similar between the SCC and ACC (Figures 2B, 2C). However, several differences were seen among the age groups. In young women, the frequency of HPV16 was higher in ACC than in SCC (75% vs. 56.3%; p<0.0001, Fisher's exact test), and the lack of HPV45, HPV31, HPV39 or HPV-negative cases and the low frequency of other HPVs in ACC is noteworthy. As expected, the frequency trends of all HPVs in SCC were very similar to the trends observed in the entire cancer sample set, since this tumor type represent 83.5% of all CC studied (compare Figures 2A and 2B). The HPV16 and HPV18 trends are different in ACC. The curves follow a U shape, with a higher frequency in the younger patients, a decrease in the middle-aged patients, then an increase in older patients. In fact, the fall of HPV16 frequency from the younger to the middle-aged patients was much higher in ACC (75.0% vs. 39.3%, p = 0.046, Fisher's exact test) than in SCC (56.3% vs. 43.9%, p = 0.01, chi-square test), although the significance was higher for the latter due to the small ACC sample size. On the other hand, the frequency of single infections with HPVs other than HPV16, HPV18, HPV45, and HPV39 including double infections followed an inverted trend compared with HPV16 and HPV18 in ACC. Interestingly, the pooled frequency of HPVs 16, 18, 45, and 39 in the older women was much higher in the ACC than in the SCC tumors (68.4% vs. 52.6%).

**Figure 4:**
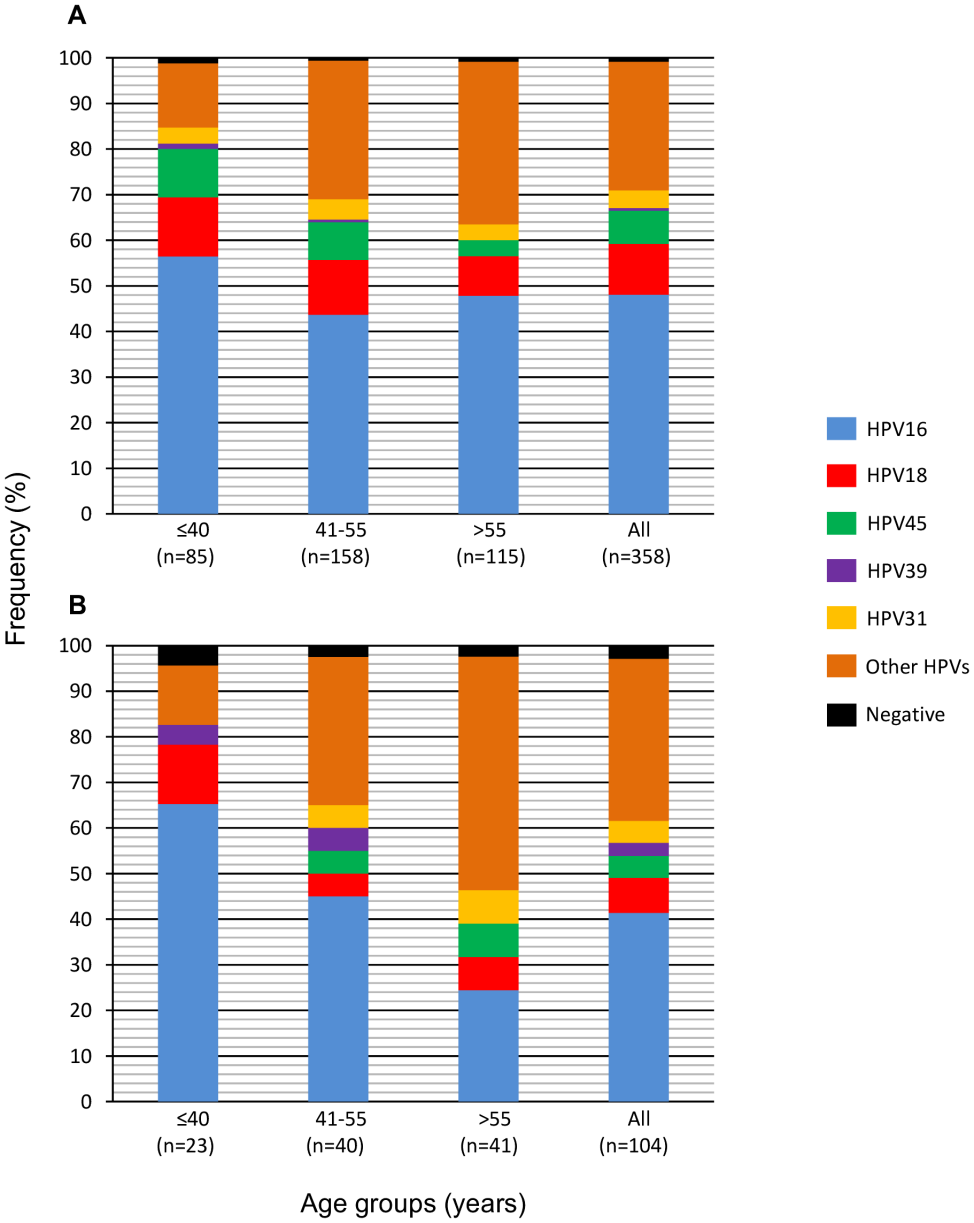
Frequencies of different HPV types in CC patients according to age and FIGO staging. The figure shows the relative frequency (%) of HPV types in CC patients grouped by age in ≤40, between 41 and 55 and>55 years old. Panel A included the patients with FIGO I/II, and panel B patients with FIGO III/IV. Bars labeled as HPV16, HPV18, HPV45, HPV31 and HPV39 include only single infections. Other HPVs group include single infection of HPV types 6, 11, 26, 33, 35, 42, 51, 52, 53, 56, 58, 59, 61, 66, 68, 69, 70, 73, 82, 39-like, 51-like, 82-like and all double infections.

Whereas in FIGO stage I/II tumors, the frequency trends of all HPV groups were very similar to the trends in the whole CC sample set (Figure 4A), in stage III/IV tumors, the HPV16 trend falls dramatically (40.8%) from the younger to the older patients, and the reverse occurs for the trend of HPVs other than HPVs 16, 18, 45, and 39 including double infections (45.5%) (Figure 4B).

### Analysis of life-style factors according to age and HPV types

We investigated whether the patients' life-style factors were associated with the distribution of the disease by age. The factors studied were age at first sexual intercourse, lifetime number of sexual partners, marital status, contraceptive use, smoking, age at first pregnancy and previous Pap screening. Each of these factors were divided into two groups, according to the risk reported for CC, the lower-risk or reference variable (labeled with an asterisk in Tables 3 and 4) and the higher-risk variable. We found a statistically significant variation in the distribution of low- and high-risk variables of age at first sexual intercourse, lifetime number of sexual partners, contraceptive use, age at first pregnancy and previous Pap screening among the age groups (Table 3). In all these but one factor (Pap screening), the frequency of high-risk variables peak in patients ≤40 yrs, then it follows a decreasing trend as increase the age range. Conversely, the frequency of the low-risk variables follows a reverse trend, it increase from patients ≤40 yrs to patients ≥55 yrs (p<0.01, Pearson chi-square; table 3). Therefore, these low-risk variables or rather the absence of high-risk variables seem to be associated with the late onset of the CC. In the case of the Pap screening, only one third of the patients reported to assist regularly to screening test and almost this proportion (∼59%) was maintained in the first two age groups (≤40 years and 41–55 years). However, in the older group it increased up to 77.6% (p<0.01, Pearson chi-square; table 3).

**Table 3:** Variation of life-style factors in CC patients grouped by age.

Life-style factor	Age Groups: n (%)				
	≤40	41–55	≥56	Total	p value^a^
**Age at first sexual intercourse** b					
≥19 yrs*	11 (10.2%)	55 (28.4%)	49 (31.8%)	115 (25.2%)	
≤18 yrs	97 (89.8%)	139 (71.6%)	105 (68.2%)	341 (74.8%)	<0.0001
Total	108 (100%)	194 (100%)	154 (100%)	456 (100%)	
**Lifetime number of male sexual partners** b					
1*	35 (32.7%)	79 (40.5%)	82 (53.2%)	196 (43%)	
≥2	72 (67.3%)	116 (59.5%)	72 (46.8%)	260 (57%)	0.003
Total	107 (100%)	195 (100%)	154 (100%)	456 (100%)	
**Marital Status**					
Married*	51 (47.2%)	89 (45.4%)	63 (40.6%)	203 (44.2%)	
Other^c^	57 (52.8%)	107 (54.6%)	92 (59.4%)	256 (55.8%)	0.51
Total	108 (100%)	196 (100%)	155 (100%)	459 (100%)	
**Contraceptive Use**					
Never*	79 (73.1%)	150 (75.8%)	137 (87.8%)	366 (79.2%)	
Ever	29 (26.9%)	48 (24.2%)	19 (12.2%)	96 (20.8%)	0.004
Total	108 (100%)	198 (100%)	156 (100%)	462 (100%)	
**Smoking**					
Never*	88 (81.5%)	155 (78.3%)	120 (76.9%)	363 (78.6%)	
Ever	20 (18.5%)	43 (21.7%)	36 (23.1%)	99 (21.4%)	0.67
Total	108 (100%)	198 (100%)	156 (100%)	462 (100%)	
**Age at first pregnancy**					
≥19 years^d^ *	42 (38.9%)	99 (50%)	99 (63.5%)	240 (51.9%)	
≤18 years	66 (61.1%)	99 (50%)	57 (36.5%)	222 (48.1%)	<0.0001
Total	108 (100%)	198 (100%)	156 (100%)	462 (100%)	
**Previous Pap Screening**					
Ever^e^ *	44 (40.7%)	81 (40.9%)	35 (22.4%)	160 (34.6%)	
Never	64 (59.3%)	117 (59.1%)	121 (77.6%)	302 (65.4%)	<0.0001
Total	108 (100%)	198 (100%)	156 (100%)	462 (100%)	

a Pearson chi-square.

b Information of six patients was missed.

c Include widowed, divorced, cohabiting, singles.

d Include nulliparous (3.4% of total cases).

e Patients that have been assisted at least once to Pap screening

*Lower-risk factor or reference factor for CC

**Table 4:** Association of life-style factors and HPV types with delayed onset of cervical cancer.

Factor	Age Groups:n (%)			
	≤40 yrs	≥41 yrs	OR (95% CI)^a^	p value
**HPV infection**				
Other HPVs^b^ *	18 (16.9%)	139 (39.7%)	2.9 (1.6–5)	2.2×10^−4^
HPVs16/18/45/39	88 (83.1%)	211 (60.3%)	1	
Total	106 (100%)	350 (100%)		
**Age at first sexual intercourse** c				
≥19 years*	11 (10.2%)	104 (29.9%)	2.5 (1.2–5.2)	0.02
≤18 years	97 (89.8%)	244 (70.1%)	1	
Total	108 (100%)	348 (100%)		
**Lifetime number of male sexual partners** c				
1*	35 (32.7%)	161 (46.1%)	1.7 (1.1–2.8)	0.03
≥2	72 (67.3%)	188 (53.9%)	1	
Total	107 (100%)	349 (100%)		
**Contraceptive Use**				
Never*	79 (73.1%)	287 (81.1%)	1.2 (0.7–2.1)	0.48
Ever	29 (26.9%)	67 (18.9%)	1	
Total	108 (100%)	354 (100%)		
**Age at first pregnancy**				
≥20 years^d^ *	42 (38.9%)	198 (55.9%)	1.5 (0.9–2.4)	0.13
≤19 years	66 (61.1%)	156 (44.1%)	1	
Total	108 (100%)	354 (100%)		
**Previous Pap Screening**				
Ever^e^ *	44 (40.7%)	116 (32.7%)	0.77 (0.48–1.2)	0.28
Never	64 (59.3%)	238 (67.3%)	1	
Total	108 (100%)	354 (100%)		

a Patient group ≤40 yrs was taken as reference group and odds ratios were calculated using a logistic regression model including all significant variables of table 3; reference variable (OR = 1), p value and 95% confidence interval are shown.

b Other HPVs includes single infections other than HPV16/18/45/39 and double infections.

c Information of six patients was missed.

d Include nulliparous (3.4% of total cases).

e Patients that have been assisted at least once to Pap screening.

*Lower-risk factor or reference factor for CC.

To investigate whether these life-style factors and HPV types other than HPV16/18/45/39 are independently associated with delayed onset of CC, we use a multivariate logistic regression (MLR) model with dichotomous outcome (age group) and explanatory variables (HPV types and life-style factors). Since the most notorious changes in the frequency of HPV types and risk factors occurred between the first two groups of age (≤40 yrs and 41–55 yrs), for this analysis we divided the patients in two age groups: ≤40 yrs and ≥41 yrs. The group of patients ≤40 yrs was taken as the reference group and the high-risk variables as the reference variables (see table 4). When these factors were included in the same model, only HPV infection with HPVs other than HPV16/18/45/39 (OR: 2.9, 95% CI: 1.6–5), age at first sexual intercourse ≥19 yrs (OR: 2.5, 95% CI: 1.2–5.2), and one lifetime sexual partner (OR: 1.7, 95% CI: 1.1–2.8) remained significantly associated with delayed onset of CC (p<0.05, Wald chi-square statistic). The overall model provided a better fit to the data over the null model (p<0.0001, Likelihood ratio test), it was fit to the data well (p>0.05, Hosmer–Lemeshow test), and the overall prediction was 76.3%, an improvement over the chance level (50%). These results indicate that these three low-risk variables are independent and contribute to the late onset of CC.

## Discussion

Although there are numerous studies of HPV frequencies in women with CC, little is known about HPV frequency according to age. In this study, we analyzed, in detail, HPV infection according to age in CC patients. The mean ages of the patients singly infected with HPV16, HPV18, HPV45, and HPV39 were at least 5 years lower than the mean age of the patients singly or doubly infected with the other HPVs. Even more interesting was the finding that the frequency trends of these groups of HPVs become completely different as the patients' ages increase. Three different trends could be identified: one for HPV16, another for HPVs 18, 45, and 39, and a third for the rest of HPVs. HPV16 follows the typical trend with two peaks as has been reported in healthy women in many studies, especially from Latin American and Asian countries [6], [12], [13]. The first and highest peak was found in the youngest women (≤35 years), followed by a decreasing trend until the ages of 55–60 years. A second peak arose at 61–65 years, followed again by a decreasing trend. The trend of HPVs 18, 45, and 39 declined similarly to the trend of HPV16, but without a second peak; it declined continuously until the end of the age intervals, instead. In contrast, the trend for the rest of the HPVs increased continuously from the youngest to the oldest women. There is one report that associates the frequency trend of high-risk HPVs in healthy women (in this case bimodal) with the bimodal frequency trend of CC in Hong Kong [6]. In the case of our population, the bimodal HPV16 trend, either in healthy [14] or cancer-stricken women (this report), does not seem to relate with the age frequency distribution of CC, because only one CC peak was found (Figure 1).

There is a great contrast in the frequency of HPVs between the extreme age groups. Whereas the pooled frequency of HPVs 16, 18, 45, and 39 is 64.7% in the whole sample, the frequency in the younger women increases to 81.5% and decreases to 54.5% in the older women. This difference (27 years) was similar, but in the opposite direction, for the frequency of the rest of HPVs; it increases from 16.7% in patients younger than 41 years to 44.2% in patients older than 55 years. The numbers in the whole sample contrast with the reported frequencies for those HPV groups in most studies, mainly from developed countries, in which the pooled frequency of HPVs 16, 18, and 45 is between 75–80% and between 20–25% for the rest of HPVs [8], [15]. However, the frequencies of HPV16 and HPV18 in this study are consistent with previous reports in Mexican populations [16]. These differences may be related to the age of patients at CC diagnosis; whereas the patients' mean age in this study was 50 years, in most developed countries is 5 to 10 years lower [17], [18]. The changes in the prevalence of the most common HPVs in women with CC according to age have been reported in populations from Europe and South America [9], [10], [19], [20]. Considering the HPVs included (HPVs 16 and 18) by the commercially available preventive vaccines, the global coverage percentage in Mexico would be 74.1% for young women and 55.8% for women older than 55 years. These percentages must be considered in order to develop better strategies for the CC prevention program in Mexico, which have to include pre- and post-menopausal women. In Mexico the Pap coverage is still low (66%) compared with higher-income countries [21]. With the introduction of the preventive vaccines in young girls is expected a significant decrease in the cervical cancer incidence [22], [23]. However, vaccinated women must continue to participate in early detection programs for CC because the vaccines can only protect against certain virus types, and it is not known for how long the immune protection against the targeted virus remains [24], [25]. On the other hand, the screening program has to be reinforced in post-menopausal women, since the high prevalence of HPV types other than those included in current preventive vaccines.

It has been shown that the most oncogenic HPVs are HPV16 and HPV18, followed by HPV45 [26], [27]. Therefore, these infections may rapidly evolve to invasive CC [8], [28]. These data could explain the higher percentage of these common HPVs in younger patients, but disagree with data from CC patients older than 55 years. These patients are 15 to 60 years older than the younger patients. It is not known whether these patients acquired the infection 10–15 years before being diagnosed with CC or were infected many years ago in their youth and the virus remained latent [29], [30], [31]. According to the HPV frequency trends observed in this study, this phenomenon could not be the same for HPV16, HPV18 and HPV45, or for the other HPVs. In the case of HPV16, its bimodal frequency curve suggests the presence of two different types of infections, which could be linked to different variants, genetic, or physiologic variations, or to differences in patients' lifestyles. There are data that support the HPV variants hypothesis. It has been demonstrated that, in Mexico, nearly 40% of HPV16 infections are due to Asian-American (AA) HPV variants AA-a and AA-c, which confer nine times more risk than the European (E) variants for CC development. Furthermore, women positive for the AA-c were on average 5 years younger than patients positive for the E and AA-a variants [32]. In addition, in vitro experiments have shown that the AA-c variant E2 gene does not repress E6/E7 transcription [33], suggesting that this variant could be more aggressive since it would shorten the time from the initial infection to the generation of an invasive cancer. These data suggest that a higher frequency of AA-c variant could also be associated with CC in the younger patients in our study. The diminished frequencies of HPVs 16, 18, and 45 with age could be also linked to lower levels of hormonal stimuli. There are consensus sequences for estrogen receptors in the control regions of these viruses that may play an important role in HPV gene regulation [34]. On the other hand, the data could suggest that, since their frequencies increase with age, some HPV16 variants, as well as the other HPVs, do not require estrogenic stimuli to replicate. However, we also cannot rule out the possibility that the immune response has an important role in the HPV distribution differences. For instance, non-common HPV types have lower replication rates [35]; therefore they could remain silent for the immune response. Moreover, it has been reported that the immune response diminishes in post-menopausal women [36]. This could produce an environment adequate for dormant HPVs or even allow for the acquisition of new HPV16 infections.

In this work we found that 2.4% of CCs only had low-risk HPVs, predominating HPV11 and HPV6. Similar percentages have been found in large samples of CC explored for HPV types [8], [37], [38], [39]. Although these viral types have low or null oncogenic potential, there are isolated reports which describe mutations in the oncogenes of these viruses that could increase their oncogenicity [40], [41]. On the other hand, this finding could also be related with the tumor sampling. There are several reports that have demonstrated CC frequently has contiguous pre-neoplastic or benign lesions, which could have low-risk HPVs [42], [43]. Because in most CC the HPV types were detected from scrapes, a detection bias may occur, and especially those low-risk types may not represent the types that actually caused cervical cancer. Finally, it is important to consider that the HPV typing methods used in this study have limitations for detecting multiple infections. The L1-based universal HPV typing method used in this study mainly detects the HPVs with the higher copy number in the sample, and the E6-based specific HPV typing method, only detects some high-risk HPV types. However, since the frequency of the double infections that we found is similar to the reported in most published cervical cancer papers [8], [27], [38], it suggests that these combined methods detected most double infections. In any case, we cannot rule out that those CCs have mixed infections, with high-risk HPVs no detected with our E6-based specific HPV typing method or having lower copy number than low-risk HPV types.

Co-infections of the HPVs16, 18 or 45 with other high-risk HPVs have been frequently reported. The high-risk HPVs other than HPV16, 18, and 45 have lower oncogenic potential. In fact, just a small percentage (<3%) of those infections progress to CINIII in a time of 10 years [26], [27]. This suggests that their potential to drive the process of an invasive cancer is low. Therefore, in the case of these co-infections in old women, the HPV16, 18 or 45 could infect the cervix belatedly and be the HPVs that actually lead the carcinogenic process.

On the other hand, as expected, low-risk sexual behavior (late age of first sexual intercourse and one lifetime sexual partner) [44], [45], and HPV types with reduced oncogenic potential (HPVs other than HPV16/18/45) were independently associated with late onset of CC. On the contrary, HPV infection with HPVs16/18/45/39, age at first sexual intercourse ≤18 yrs and more than one lifetime sexual partner were associated to early onset of CC. However, to better define the effect of life-style factors and HPV types on the onset of CC, a case-control study is needed.

Finally, CC is a rare complication of the viral infection, most infections are transient and do not evolve into neoplastic lesions [25], [26]. This suggest that HPV infection alone does not cause CC, and other factors, such as duplications, deletions, point mutations, or epigenetic regulation of host genes, contribute to the development of invasive cancer. In this scenario, it is expected that tumors positive for HPVs other than HPVs 16, 18, and 45 evolve to an invasive state through the accumulation of a higher number of genomic alterations than those needed for tumors positive for HPVs 16, 18, or 45. In fact, results from next-generation sequencing of 325 of the 462 CCs examined in this paper showed that the number of mutations in cancer genes increase with the age of patients (Mike Dean, personal communication).

Therefore, these data collectively suggest that in a fraction of CC in women older than 55 years, especially SCC with advanced clinical stages, the role of HPV seems to be secondary, and other factors could play the principal role in the process of carcinogenesis.

## Methods

### Ethics Statement

The study protocol was approved by the Scientific and Ethics Committees of the Hospital General de Mexico (approval number DIC/03/311/04/051) and was performed in accordance with the ethical principles described in the 1964 Declaration of Helsinki. Informed written consent was obtained from all participants prior to their inclusion in the study.

### Subjects and Samples

A cross-sectional study was done including a total 503 patients diagnosed with CC at the Department of Oncology of the Hospital General de Mexico in Mexico City. Patients were referred from the Outpatient, Emergency or Gynecology Departments, and from the CC screening program. Most of the patients come from the Metropolitan area (Mexico City and State of Mexico) and nearby states. All patients are Mexican-mestizo and have an important Amerindian genetic background [46]. The Hospital attends patients without social security and the CC screening program serves on average 100 women per day. However, two thirds of patients recruited in the present study occasionally or never have attended a screening program. The inclusion criteria of the study were clinical diagnosis of invasive cervical cancer at the Oncology Department, no previous treatments, born and reside in Mexico and have a Mexican ancestry of two generations backward. Patients that fulfill the inclusion criteria were sequentially recruited during the periods from November 2003 through April 2005 and January 2006 through July 2007 and represented about 80% of the patients diagnosed with CC during this period. All patient subjects received a complete clinical evaluation by an experienced oncologist. Tumor staging was carried out according to the last international revised protocol for gynecological cancer [47]. Two biopsy samples were taken from the tumors during colposcopy examinations. One sample was divided into two equal parts: one part was fixed in buffered formalin for morphological analysis and the other part, together with the second biopsy sample, was snap-frozen on dry ice and stored at −80°C until analysis. From these patients, 41 were excluded because the biological samples were of insufficient quality, or because their cases were confirmed as CIN2/3 or in situ carcinomas by consensus of three pathologists. These exclusions left 462 CC patients with available biological samples for the analysis. For HPV detection and typing, a scrape from the endocervix and ectocervix was collected with a cytobrush, and the cells were suspended in a vial with extraction buffer and stored at −20°C until analysis. Of the patients analyzed, 179 (38.7%), 179 (38.7%), 80 (17.3%) and 24 (5.2%) had Federation of Gynecology and Obstetrics (FIGO) stage I, II, III, and IV tumors, respectively. Most of the tumors analyzed were squamous cell carcinoma (SCC), with a frequency of 386 (83.5%), followed by adenocarcinoma (ACC), adenosquamous cell carcinoma (ASCC), and undifferentiated (IND), with a frequency of 63 (13.6%), 7 (1.5%), and 6 (1.4%), respectively.

### Detection and HPV typing

DNA was extracted and purified from cervical scrapes and biopsy specimens using the PureLink genomic DNA kit (Invitrogen, Carlsbad, CA, USA) and maintained at −20°C until analysis. HPV detection was performed on all cervical scrapes, and when the internal control or HPV signal was weak (n = 44), or the samples yielded negative results for HPV (n = 21), the DNA extracted from the biopsy sample was also examined. In these cases, the DNA results obtained from the biopsy sample determined the presence or absence of HPV. In addition, to validate the analyses done on the scrapes, HPV detection and typing were carried out on another 100 biopsy samples, in which the signals of the paired scrapes were of good quality. The agreement between these parallel experiments was 98% (kappa = 0.971, 95% CI: 0.931–1.00). The HPV detection was performed by PCR using universal primers located in HPV L1 genes *MY09*/*MY11*, *GP5+*/*6+*, and *L1C1*, as described previously [48], [49], [50]. The *HBB* gene was used as an internal control to assess the quality of the DNA. The HPV types were identified by sequencing the amplified bands in positive samples using the fluorescent cycle-sequencing method (BigDye Terminator Ready Reaction Kit; Applied Biosystems, Carlsbad, CA). Sequence analysis was performed using an ABI PRISM 3130×l Genetic Analyzer system (Applied Biosystems, Carlsbad, CA). Each band sequenced from the HPV-positive samples was analyzed with the FASTA sequence similarity tool [51]. The average identity percentage of HPV types detected was 98.7% (range: 78–100%) when compared to the reference sequences. All but 4 HPVs had a sequence similarity>90%. The sequences of these HPV-like types have less than 90% identity with the closer HPV reference type: HPV39-like (n = 1, 85%), HPV51-like (n = 2, 78% and 80%), and HPV82-like (n = 1, 80%).

In addition, all samples were assayed using E6/E7-specific primers for HPV16 (forward 5′-ATTAGGTGTATTAACTGTCAAA -3′ and reverse 5′-TTCTGCTTGTCCAGCTGG-3′) and HPV18 (forward 5′-CGACGATTTCACAACATAGC-3′ and reverse 5′-TCACACTTACAACACATACAC-3′), setting the annealing temperature 5°C below the Tm. Briefly, 500 ng of sample DNA was added to achieve a final volume of 25 µL containing 20 mM Tris-HCl (pH 8.8), 2 mM MgCl_2_, 50 mM KCl, 1 mM DTT, 200 µM of dATP, dTTP, dGTP and dCTP, 1 µM of each oligonucleotide, and 1 U of Taq DNA polymerase (Invitrogen, Camarillo, CA, USA). After denaturing for 2 minutes at 94°C, the reaction was carried out over 40 cycles in a DNA Thermal Cycler (Gene Amp PCR System 9700; Applied Biosystems, Carlsbad, CA). Station 1 was set at 94°C for 30 seconds, station 2 at 52°C for 30 seconds, and station 3 at 72°C for 30 seconds. After this treatment, the reaction was kept at 72°C for another 7 minutes. After the PCR was completed, 5-µL aliquots were electrophoresed for 1 hour at 120 V in 2% agarose gels with 0.3 µg/mL ethidium bromide. The specific fragments were detected using a UV transilluminator (Pharmacia, USA) according to the positions of the positive controls and the molecular weight markers in the gel. The HPV16 and HPV18 bands were sequenced. In addition to HPV16 and HPV18 types, other viral types phylogenetically related to them could also be detected (HPVs 31, 33, 52 and 58 related to HPV16, and HPVs 39, 45, 59 and 68 related to HPV18). When different viral types were identified with the specific and universal primers, those samples were considered doubly infected.

### Data Analysis

The HPV frequencies were compared using the chi-square test, Yates' adjusted, or Fisher's exact test. For the analysis of the proportion trends among the groups, the Cochran-Armitage trend test was used. The mean age of the patients was compared using the t-Student or one-way ANOVA test. The Spearman correlation was used to evaluate the HPV frequency trend among the 5-year interval groups. The effect of life-style factors on the distribution of CC patients by age groups was assessed using the Pearson chi-square test. To investigate whether the life-style factors and HPV-types groups are independently associated with delayed onset of CC, we use a MLR model with dichotomous outcome (age group) and explanatory variables (HPV types and life-style factors). Differences were considered significant with a p value <0.05. All statistical tests were carried out using SigmaPlot, XLSTAT, and SPSS ver. 17 software.
